# Low Anterior Resection Syndrome (LARS) after Surgery for Rectal Cancer: An Inevitable Price to Pay for Survival, or a Preventable Complication?

**DOI:** 10.3390/jcm12185962

**Published:** 2023-09-14

**Authors:** Edoardo Maria Muttillo, Alice La Franca, Alessandro Coppola, Francesco Saverio Li Causi, Marzia Checchelani, Alice Ceccacci, Giorgio Castagnola, Giovanni Maria Garbarino, Mattia Falchetto Osti, Genoveffa Balducci, Paolo Mercantini

**Affiliations:** 1 Department of Medical Surgical Science and Translational Medicine, Sant’Andrea Hospital, Sapienza University of Rome, 00191 Rome, Italy; alicelafranca@gmail.com (A.L.F.); licausi.1643056@studenti.uniroma1.it (F.S.L.C.); checchels@gmail.com (M.C.); alicececcacci@libero.it (A.C.); giorgiocastagnola@hotmail.com (G.C.); genoveffa.balducci@uniroma1.it (G.B.); paolo.mercantini@uniroma1.it (P.M.); 2 Department of Surgery, Sapienza University of Rome, Viale Regina Elena 291, 00161 Rome, Italy; coppola.chirurgia@gmail.com; 3 Department of General Surgery, Sant’Eugenio Hospital, 00144 Rome, Italy; giovannimaria.garbarino@uniroma1.it; 4 Radiotherapy Oncology, Sant’Andrea Hospital, Sapienza University of Rome, 00191 Rome, Italy; mattiafalchetto.osti@uniroma1.it

**Keywords:** rectal cancer, low anterior resection syndrome, rectal surgery, LARS, ileostomy

## Abstract

Background: Rectal cancer is frequent and often treated with sphincter-saving procedures that may cause LARS, a syndrome characterized by symptoms of bowel disfunction that may severely affect quality of life. LARS is common, but its pathogenesis is mostly unknown. The aim of this study is to assess the incidence of LARS and to identify potential risk factors. Methods: We performed an observational retrospective single center analysis. The following data were collected and analyzed for each patient: demographics, tumor-related data, and intra- and peri-operative data. Statistical analysis was conducted, including descriptive statistics and multivariate logistic regression to identify independent risk factors. Results: Total LARS incidence was 31%. Statistically significant differences were found in tumor distance from anal verge, tumor extension (pT and diameter) and tumor grading (G). Multivariate analysis identified tumor distance from anal verge and tumor extension as an independent predictive factor for both major and total LARS. Adjuvant therapy, although not significant at univariate analysis, was identified as an independent predictive factor. Time to stoma closure within 10 weeks seems to reduce incidence of major LARS. Conclusions:bold LARS affects a considerable portion of patients. This study identified potential predictive factors that could be useful to identify high risk patients for LARS.

## 1. Introduction

Colorectal cancer is the third most commonly diagnosed cancer in the world, and the second most common cause of cancer death [1]. Total mesorectal excision (TME) and neoadjuvant radiotherapy are considered the cornerstones of treatment for potentially curable rectal cancer, and their introduction has radically improved oncological outcomes, both in terms of increased survival and reduction in permanent stoma rates [2,3]. Although surgical treatment has significantly improved, patients who undergo a sphincter-saving procedure may experience symptoms and consequences of bowel dysfunction that can severely affect their quality of life [4,5]. These symptoms include variable and unpredictable bowel function, altered stool consistency or frequency, repeated painful evacuations, emptying difficulties, urgency, incontinence, and soiling [6]. The term low anterior resection syndrome (LARS) has been adopted to refer to this syndrome [4]; however, its definition has only recently been standardized based on international consensus [6]. LARS is consistently linked to a decrease in the quality of life. As access to treatment for rectal cancer improves, LARS increasingly becomes a significant burden of disease [7]. Surgical trauma to the anal sphincter complex, colonic denervation, reduced rectal capacity and compliance, radiotherapy-induced fibrosis, fecal diversion and, more recently, also altered colonic mobility, have been identified as potential risk factors for LARS [8]; however, their exact role is unknown. The development of a validated patient-reported outcome measure (LARS score) has improved the standardization of reporting and prevalence of LARS defined using this score is reported to be 41% (95% CI, 34–48%) [9]. The aim of this study is to assess the incidence of LARS according to LARS scores in patients who underwent sphincter-saving resection in our center, and to identify possible predictive factors.

## 2. Materials and Methods

We performed an observational retrospective single center analysis.

**Figure 1:**
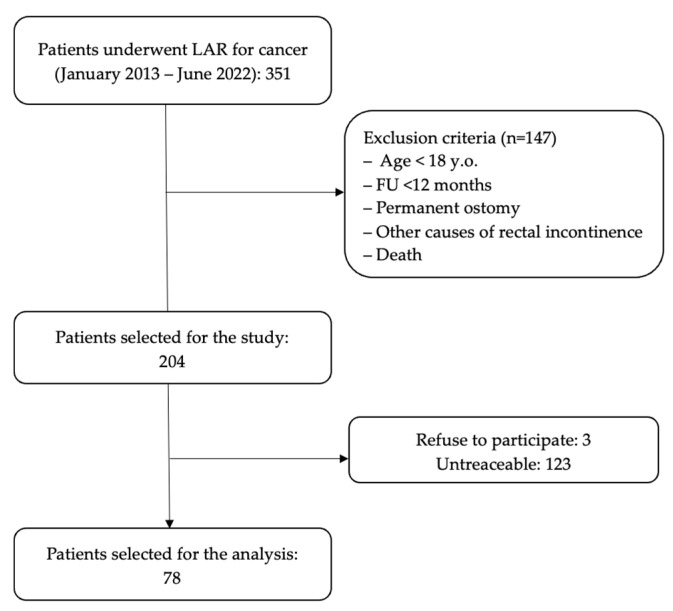
Flowchart.

Patients who underwent Low Anterior Resection (LAR) for cancer at the General Surgery Department at Sant’Andrea Hospital in Rome from January 2013 to June 2022 were selected. Exclusion criteria were age < 18 years, LAR for benign diseases, follow up <12 months, permanent ostomy, other evident causes of fecal incontinence (for instance, advanced dementia) and death. Out of the 351 patients initially considered, 147 were excluded due to not meeting the inclusion criteria, 123 could not be located, and 3 declined to participate. Ultimately, a total of 78 patients were chosen for the analysis (Figure 1).

The following data were collected and analyzed for each patient: demographics (age, sex), tumor-related data (TNM stage, distance from anal verge, grading), and intra- and perioperative data (American Society of Anesthesiologists (ASA) score, surgical technique, operative time, ileostomy, time to stoma closure, neoadjuvant and/or adjuvant therapy, morbidity according to Clavien-Dindo score) [10].

Among the 78 patients, 30 females and 48 males, median age was 65 years (range 57–73). The entire population underwent low anterior resection with anastomosis and protective ileostomy was performed in 34% of cases. The distance of the tumor from the anal verge was ≥10 cm in 49%, between 5 and 10 cm in 38% and ≤5 cm in 13%.

### 2.1. Statistical Analysis

Continuous variables are presented as median and interquartile range (IQR), while categorical variables are expressed as units and percentages. Descriptive statistics were used to summarize information relevant to the study. The differences between groups were analyzed using Wilcoxon rank sum test for continuous variables, and Pearson’s Chi-squared test or Fisher’s exact test, as appropriate, for categorical variables. A multivariate binomial logistic regression was developed to identify independent predictors of outcomes. Significance was accepted at *p* < 0.05. All statistical analyses were performed using R (version 4.3.1; R Foundation for Statistical Computing, Vienna, Austria).

### 2.2. LARS Score

**Figure 2:**
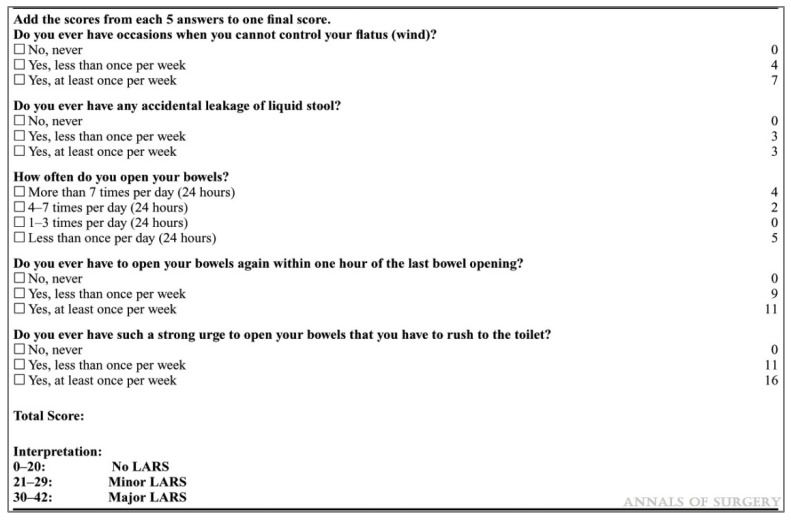
LARS questionnaire by Emmertsen et al., *Annals of Surgery*, 2012 [9].

Each patient was reached telephonically and subjected to LARS questionnaire, consisting of five questions as shown in Figure 2. The allocated points per question were added together to give a final LARS score between 0 and 42. Finally, the population was divided into three categories [9]:

- No LARS (L0): 0–20;
- Minor LARS (mL): 21–29;
- Major LARS (ML): 30–42.

## 3. Results

In the examined population, 30 patients (38%) were females and 48 (62%) were males, with a median age of 65 years (IQR 57, 73). A total of 28 patients (36%) had an American Society of Anesthesiologists score > 2.

The distance of the tumor from the anal verge was ≥10 cm in 38 patients (49%) and between 5 and 10 cm in 30 patients (38%). Ten patients (13%) underwent ultra-low resection for a tumor localized at ≤5 cm from the anal verge (Table 1).

Each patient underwent LAR with anastomosis, 53 (68%) with open approach and 25 (32%) with laparoscopic approach. Median operative time was 210 min (IQR 180, 248). Ostomy formation was performed on a total of 29 patients, which accounts for 37% of the study’s participants. Protective temporary ileostomy was performed in 27 cases, while in two patients, a salvage ileostomy was created due to anastomotic leak. Finally, 49 patients (63%) were discharged without ileostomy.

Moreover, in 14 cases (17.95%), other intraoperative procedures were associated (two single liver metastasectomies, one splenectomy, one colostomy reversal, three ovariectomies, one hysterectomy with bilateral ovariectomy, one ureteral stent for intraoperative damage, three cholecystectomies, one umbilical hernia repair).

The overall complication rate was 17% (12 patients), with 7 grade I-II and 1 grade IIIa according to Clavien-Dindo classification [10]. A total of four patients underwent emergency surgery, two for anastomotic leak, one for a fistula and one for rectal bleeding (grade IIIb).

A total of 28 patients (36%) underwent neoadjuvant radiotherapy, 24 (86%) with long-course protocol and four (14%) with short-course protocol. Finally, eight patients (10%) underwent adjuvant therapy (Table 2).

The total LARS (TL) rate was 31% (n = 24), with 17% of patients (n = 13) manifesting the symptoms of minor LARS and 14% (n = 11) of major LARS.

By univariate analysis, the following predictive factors for LARS were found to be statistically significant: tumor distance from anal verge (*p* = 0.041), tumor extension considered both as tumor diameter (*p* = 0.013) and pathological T stage (*p* = 0.007).

Finally, a significant difference in tumor grading (G) was present in patients with ML compared to no LARS (*p* = 0.038) (Table 3).

Multivariate analysis confirmed the role of tumor distance from anal verge as an independent predictive factor for both ML and TL (*p* = 0.006), with tumors located at 5–10 cm presenting the highest risk of LARS development (OR 5.58). Tumor extension, both in terms of diameter and T stage, was also found to be an independent predictive factor for both minor and total LARS (*p* = 0.015). Finally, although not significant at univariate analysis, adjuvant therapy was identified as an independent predictive factor for minor, major and total LARS (*p* = 0.004) (Table 4).

## 4. Discussion

Patients who underwent low anterior resection with TME, which remains the gold standard treatment for rectal cancer [11,12], can develop a functional anorectal alteration known as low anterior resection syndrome.

Bearing in mind the strong negative impact on patients’ quality of life, the aim of this study was to calculate incidence of major and minor LARS, and to identify possible predictive factors.

Our results showed a TL rate of 31% (26/78 patients), with a 14% rate of major LARS, which seems far below values reported in literature that can reach up to 40% for ML and 65% for TL [13].

In this study, the following factors emerged as predictive for the development of LARS: tumor size (T stage), adjuvant therapy and distance from the anal verge.

Increased tumor size in itself, calculated in centimeters, did not translate into increased rates of LARS, but tumor size as pT stage showed an impact on LARS. While, as might be expected, as pT increased, so did the risk of developing TL (*p* = 0.015) and mL (*p* = 0.003); a surprising finding was the increase in TL shown in the pT2 group (OR 9.05) compared to pT3 (OR 0.91). In the opinion of this research group, though, this finding can be attributed to the nonnegligible fraction within the pT2 group of patients downstaged following neoadjuvant therapy which, although not found to be significant on our series, is recognized in the literature as a risk factor for LARS [9,14,15,16,17,18,19].

Li et al. conducted a meta-analysis in 2022, including 33 studies and involving 17,917 patients. The study concluded that neoadjuvant therapy emerged as an independent risk factor for the significant development of major LARS, with an odds ratio of 3.09 (*p* < 0.001) [20].

Similar results were obtained by Rui Sun et al. in 2021 [21]; total incidence of ML was 44% and long course neoadjuvant radiotherapy has shown OR 2.89 (*p* < 0.01).

This role of oncological therapy on the development of LARS in our series was confirmed by analysis of the adjuvant therapy effect. In fact, a combination of adjuvant chemotherapy and radiotherapy has been shown to be a risk factor for all subgroups (total, minor and major LARS).

In the univariate analysis, tumor grading played a significant role, revealing that patients with less differentiated tumors were more prone to experiencing major LARS development (38% of ML among G3 tumors versus 0% G0-G1 and 5.9% G2).

However, on multivariate analysis, the significance was lost probably because the role of grading is to be regarded more as a non-independent risk factor (as lower differentiation is often related to more aggressive tumors and may be associated with larger size and more advanced stages more likely to require oncological therapies; all of which are risk factors for LARS, as we have already discussed).

A distance from anal verge of between 10 and 15 cm was confirmed as protective factor for development of TL (OR 0.19) and ML (OR 0.46), in accordance with global literature [22,23]. Interestingly, we found high rate of TL (40%, OR 5.58) and ML (31%, OR >1000) in patients with tumors of medium rectum (between 5 and 10 cm), probably due to the closeness of this area to the hypogastric plexus nerve that can be damaged during surgery [24]. Sturiale et al., in a retrospective analysis published in 2017 including 93 patients, had similar results [19].

In this scenario, transanal TME (TaTME), which has its primary indication in the middle rectum cancer, could play a role, although the literature at the moment has not shown a difference in LARS rates between TaTME and laparoscopic TME [25].

As for the last factor considered, the presence of ileostomy was associated with a higher rate of both total LARS (41% vs. 24%) and major LARS (29% vs. 9.8%). However, these findings have shown a trend that is not statistically significant (*p* = 0.12 for TL and *p* = 0.08 for ML), probably due to the small sample size under study.

**Figure 3:**
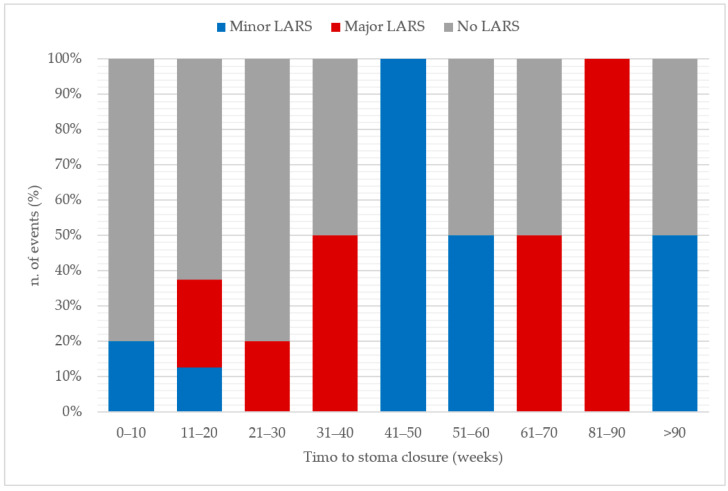
Time to stoma closure and incidence of LARS.

Other interesting data are the analysis of interval of time to stoma closure, which showed that patients with late stoma closure are more likely to develop LARS (medians to stoma closure: 43 weeks for LARS patients versus 19 weeks for no LARS group) and major LARS (39 weeks for ML group versus 19 for no LARS group). As shown in Figure 3, the best time for stoma closure appears to be within 10 weeks, even though this finding certainly needs to be validated by subsequent studies (*p* = 0.11) on a larger patient sample.

Therefore, the risk of developing LARS in patients undergoing anterior rectal resection for cancer may depend on nonmodifiable, partially modifiable, and modifiable factors. The non-modifiable factors are tumor-related: distance from the anal margin < 10 cm, larger dimension of the tumor (≥pT2) and low degree of differentiation (G3) (with the latter two factors probably related to each other). Thus, the goal must be to identify these patients to properly stratify risk and subject them to close functional follow-up. In this light, Yan et al. have developed a nomogram to stratify patients according to the risk of developing LARS that will require further data to be validated but may be a good starting point [26].

On the other hand, factors that can only be partially modified are those that involve treatment choices with an impact on survival: adjuvant therapy and ileostomy.

On this last point, in this study group’s opinion, correct indication remains fundamental. Although the literature has often focused on the burden of ileostomy in terms of quality of life and psychological impact on patients, it now appears necessary to start asking how much temporary bowel defunctionalization that derives from ileostomy exposes patients to the risk of developing functional alterations that persist even when the ostomy is closed. Indeed, if we examine the fact that ostomy seems to not act as a protective factor for anastomotic leak [27,28,29], but has to be considered a safety device in patients with anastomotic leak [30], the excess of ostomies performed becomes evident.

The main fully modifiable factor that was the object of our study, however, was the time of ileostomy closure. An early closure seems to reduce the rate of patients developing total LARS and major LARS, and this finding seems to be confirmed by the few existing studies in the literature [14,31].

Finally, to compare our findings with those presented in the literature, Parnasa et al. [32] published a retrospective single-center study of 240 patients in 2022, obtaining results similar to ours: a smaller distance from the anal verge, neoadjuvant radiotherapy and TME (versus partial mesorectal excision and, therefore, a more conservative approach on the hypogastric nerves) resulted as independent prognostic factors for LARS. He et al. in 2022 [33] conducted a post hoc analysis on 327 patients subjected to chemotherapy followed by sphincter-saving proctectomy. Long-course neoadjuvant radiation, height of anastomosis and anastomotic leak (not analyzed by our study) were reported as predictive factors for the development of ML.

On the other hand, data pertaining to early ileostomy closure are still poor and controversial, and need further validation.

This study has several limitations: first, the small sample size (resulting from the notable number of patients found to be untraceable). Second, the LARS score is, by definition, based on the symptomatology reported by patients and, therefore, does not allow excluding other causes of incontinence and functional intestinal disorders. Finally, the retrospective nature of the study requires the need to confirm the results obtained in the context of a prospective study.

## 5. Conclusions

While patients who undergo surgery for rectal cancer often achieve favorable survival outcomes, it is important to acknowledge that the impact on their quality of life remains significant. Specifically, low anterior resection syndrome affects a considerable portion of patients. Within our series, the incidence of LARS was noted to be 31%. This study further pinpointed potential predictive factors. Among these, certain factors are modifiable, such as the closure of ileostomies within a 10-week timeframe. Additionally, there are non-modifiable factors (such as distance from the anal margin, pT stage, and adjuvant therapy) that play a crucial role in identifying patients at risk of LARS development. This recognition is essential for guiding these patients toward more focused functional follow-up, ultimately enhancing their overall care.

## Figures and Tables

**Table 1:** General features of study population.

Parameter	Category	Value (n = 78) Median (IQR) or n (%)
Age, years		65 (57, 73)
Sex	Female	30 (38%)
	Male	48 (62%)
ASA	I	4 (5%)
	II	46 (59%)
	III	28 (36%)
	IV	0 (0%)
Distance from anal verge	0–5 cm	10 (13%)
	5–10 cm	30 (38%)
	10–15 cm	38 (49%)
TNM Stage	0	5 (6.4%)
	1	20 (26%)
	2a	14 (18%)
	2b	3 (3.8%)
	3a	6 (7.7%)
	3b	21 (27%)
	3c	6 (7.7%)
	4a	3 (3.8%)

ASA: American Society of Anesthesiologists score, IQR: interquartile range.

**Table 2:** Intraoperative and perioperative features.

Parameter	Category	Value (n = 78) Mean (IQR) or n (%)
Surgical technique	Open	53 (68%)
	VLS	25 (32%)
Operative time, min	Median (IQR)	210 (180, 248)
Ileostomy	Yes	29 (37%)
	No	49 (63%)
Stoma closure, weeks	Median (IQR)	25 (13, 51)
nRT	LCRT	24 (31%)
	SCRT	4 (5%)
	No	50 (64%)
Adjuvant therapy	CHT	6 (8%)
	RT	1 (1%)
	No	69 (90%)
Morbidity	Yes	11 (17%)
	No	58 (83%)
Clavien-Dindo Score	1	3 (4%)
	2	4 (6%)
	3a	1 (1%)
	3b	4 (6%)
LARS Score	No LARS	54 (69%)
	Minor LARS	13 (17%)
	Major LARS	11 (14%)

nRT: neoadjuvant radiotherapy, LCRT: long course radiotherapy, SCRT: short course radiotherapy, LARS: low anterior resection syndrome, IQR: interquartile range.

**Table 3:** Factors associated with LARS. Bold format dates mean the statistically significant data.

Parameter		Total LARS			Minor LARS			Major LARS		
		L0 (n = 54)	TL (n = 24)	*p* Value	L0 (n = 54)	mL (n = 13)	*p* Value	L0 (n = 54)	ML (n = 11)	*p* Value
Age, years	Median (IQR)	67(57, 75)	65 (55, 68)	0.159	67 (57, 75)	62 (53, 66)	0.172	67 (57, 75)	65 (56, 69)	0.446
Sex, n (%)	Female	18 (60%)	12 (40%)	0.163	18 (72%)	7 (28%)	0.209	18 (78%)	5 (22%)	0.500
	Male	36 (75%)	12 (25%)		36 (86%)	6 (14%)		36 (86%)	6 (14%)	
ASA, n (%)	1	2 (50%)	2 (50%)	0.315	2 (67%)	1 (33%)	0.221	2 (67%)	1 (33%)	0.717
	2	30 (65%)	16 (35%)		30 (75%)	10 (25%)		30 (83%)	6 (17%)	
	3	22 (79%)	6 (21%)		22 (92%)	2 (8%)		22 (85%)	4 (15%)	
Distance from anal verge, n (%)	0–5 cm	6 (60%)	4 (40%)	0.180	6 (67%)	3 (33%)	0.515	6 (86%)	1 (14%)	**0.041**
	5–10 cm	18 (60%)	12 (40%)		18 (82%)	4 (18%)		18 (69%)	8 (31%)	
	10–15 cm	30 (79%)	8 (21%)		30 (83%)	6 (17%)		30 (94%)	2 (6%)	
Tumor dimension, cm	Median (IQR)	3.15 (2.20, 4.50)	2.50 (1.85, 3.00)	0.052	3.15 (2.20, 4.50)	2.50 (2.00, 2.50)	**0.013**	3.15 (2.20, 4.50)	2.50 (1.78, 4.88)	0.752
pT, n (%)	T0	2 (67%)	1 (33%)	0.107	2 (100%)	0 (0%)	**0.007**	2 (67%)	1 (33%)	0.323
	Tis	1 (50%)	1 (50%)		1 (50%)	1 (50%)		1 (100%)	0 (0%)	
	T1	6 (67%)	3 (33%)		6 (86%)	1 (14%)		6 (75%)	2 (25%)	
	T2	9 (50%)	9 (50%)		9 (53%)	8 (47%)		9 (90%)	1 (10%)	
	T3	31 (84%)	6 (16%)		31 (94%)	2 (6.1%)		31 (89%)	4 (11%)	
	T4a	4 (57%)	3 (43%)		4 (100%)	0 (0%)		4 (57%)	3 (43%)	
	T4b	1 (50%)	1 (50%)		1 (50%)	1 (50%)		1 (100%)	0 (0%)	
pN, n (%)	N0	30 (68%)	14 (32%)	0.168	30 (79%)	30 (79%)	0.104	30 (83%)	30 (83%)	0.309
	N1	19 (79%)	5 (21%)		8 (21%)	8 (21%)		6 (17%)	6 (17%)	
	N2	5 (50%)	5 (50%)		19 (90%)	19 (90%)		19 (86%)	19 (86%)	
G, n (%)	G0	2 (100%)	0 (0%)	0.189	2 (100%)	0 (0%)	>0.999	2 (100%)	0 (0%)	**0.038**
	G1	3 (100%)	0 (0%)		3 (100%)	0 (0%)		3 (100%)	0 (0%)	
	G2	32 (78%)	9 (22%)		32 (82%)	7 (18%)		32 (94%)	2 (5.9%)	
	G3	8 (53%)	7 (47%)		8 (80%)	2 (20%)		8 (62%)	5 (38%)	
Surgical Technique, n (%)	Open	36 (68%)	17 (32%)	0.716	36 (78%)	10 (22%)	0.740	36 (84%)	7 (16%)	>0.999
	VLS	18 (72%)	7 (28%)		18 (86%)	3 (14%)		18 (82%)	4 (18%)	
Operative time, min	Median (IQR)	210 (180, 255)	208 (170, 229)	0.249	210 (180, 255)	195 (155, 225)	0.131	210 (180, 255)	210 (193, 235)	0.854
Ileostomy	Yes	17 (59%)	12 (41%)	0.118	17 (77%)	5 (23%)	0.745	17 (71%)	7 (29%)	0.083
	No	37 (76%)	12 (24%)		37 (82%)	8 (18%)		37 (90%)	4 (10%)	
Time to stoma closure, weeks	Median (IQR)	19 (12, 26)	43 (14, 71)	0.106	19 (12, 26)	46 (13, 51)	0.410	19 (12, 26)	39 (22, 74)	0.105
Neoadjuvant therapy	LCRT	16 (67%)	8 (33%)	0.642	16 (80%)	4 (20%)	0.201	16 (80%)	4 (20%)	0.815
	SCRT	2 (50%)	2 (50%)		2 (50%)	2 (50%)		2 (100%)	0 (0%)	
	No	36 (72%)	14 (28%)		36 (84%)	7 (16%)		36 (84%)	7 (16%)	
Adjuvant therapy	CHT	4 (67%)	2 (33%)	0.608	4 (67%)	2 (33%)	0.672	4 (100%)	0 (0%)	0.327
	RT	1 (100%)	0 (0%)		1 (100%)	0 (0%)		1 (100%)	0 (0%)	
	CHT + RT	0 (0%)	1 (100%)		0	0		0 (0%)	1 (100%)	
	No	48 (70%)	21 (30%)		48 (81%)	11 (19%)		48 (83%)	10 (17%)	

**Table 4:** Multivariate analysis of factors associated with LARS. Bold format dates mean the statistically significant data.

**Parameter**		**Total LARS**			**Minor LARS**			**Major LARS**		
		**OR**	**95% CI**	***p* Value**	**OR**	**95% CI**	***p* Value**	**OR**	**95% CI**	***p* Value**
Distance from anal verge	0–5 cm	-	-	**0.006**	-	-	0.9	-	-	**0.002**
	5–10 cm	5.58	0.65, 77.9		0.99	0.02, 52.0		>1000	0.00, >1000	
	10–15 cm	0.19	0.01, 2.33		0.35	0.00, 23.4		0.46	0.00, >1000	
Tumor dimension, cm	Median	0.60	0.35, 0.94	**0.025**	0.13	0.01, 0.44	**<0.001**	0.76	0.27, 1.90	0.5
pT	T0	-	-	**0.015**	-	-	**0.003**	-	-	0.2
	T1	0.91	0.03, 40.2		>1000	0.00, NA		0.21	0.00, 16.5	
	T2	9.05	0.36, 464		>1000	0.00, NA		0.52	0.00, 71.1	
	T3	0.91	0.04, 33.8		>1000	0.00, NA		0.36	0.01, 20.5	
	T4a	18.8	0.31, >1000		0.00	0.00, >1000		>1000	0.00, >1000	
	T4b	>1000	0.00, NA		>1000	0.00, NA		>1000	0.00, >1000	
	Tis	14.6	0.19, >1000		>1000	0.00, NA		0.57	0.00, >1000	
pN	N0	-	-	0.13	-	-	0.6	-	-	0.3
	N1a	0.82	0.08, 6.31		0.00			4.60	0.29, 102	
	N1b	0.26	0.01, 2.88		8.02	0.02, >1000		0.00	0.00, >1000	
	N1c	0.00	-		0.00	-		0.00	-	
	N2a	9.74	0.46, 395		14.1	0.06, >1000		0.94	0.00, >1000	
	N2b	0.00	-		0.00	-		0.00	-	
Neoadjuvant therapy	No	-	-	0.3	-	-	0.066	-	-	0.6
	LCRT	0.19	0.02, 1.49		0.03	0.00, 2.90		0.28	0.02, 4.31	
	SCRT	0.27	0.01, 8.25		13.4	0.01, >1000		0.51	0.00, >1000	
Adjuvant Therapy	No	-	-	**0.004**	-	-	**0.012**	-	-	**0.007**
	CHT	0.57	0.03, 7.14		>1000	4.21, >1000		0.00	0.00, >1000	
	RT	0.00	-		0.00	-		0.05	0.00, >1000	
	CHT + RT	>1000	0.00, NA		-	-		>1000	0.00, >1000	

OR: odds ratio, CI: confidence interval, LCRT: long course radiotherapy, SCRT: short course radiotherapy.

## Data Availability

Referring to corresponding author.

## References

[1] Sung H., Ferlay J., Siegel R.L., Laversanne M., Soerjomataram I., Jemal A., Bray F. (2021). Global Cancer Statistics 2020: GLOBOCAN Estimates of Incidence and Mortality Worldwide for 36 Cancers in 185 Countries. CA Cancer J. Clin..

[2] Heald R.J., Ryall R.D. (1986). Recurrence and Survival after Total Mesorectal Excision for Rectal Cancer. Lancet.

[3] van Gijn W., Marijnen C.A.M., Nagtegaal I.D., Kranenbarg E.M.-K., Putter H., Wiggers T., Rutten H.J.T., Påhlman L., Glimelius B., van de Velde C.J.H. (2011). Preoperative Radiotherapy Combined with Total Mesorectal Excision for Resectable Rectal Cancer: 12-Year Follow-up of the Multicentre, Randomised Controlled TME Trial. Lancet Oncol..

[4] Bryant C.L.C., Lunniss P.J., Knowles C.H., Thaha M.A., Chan C.L.H. (2012). Anterior Resection Syndrome. Lancet Oncol..

[5] Keane C., Wells C., O’Grady G., Bissett I.P. (2017). Defining Low Anterior Resection Syndrome: A Systematic Review of the Literature. Color. Dis..

[6] Keane C., Fearnhead N.S., Bordeianou L.G., Christensen P., Basany E.E., Laurberg S., Mellgren A., Messick C., Orangio G.R., Verjee A. (2020). International Consensus Definition of Low Anterior Resection Syndrome. Color. Dis..

[7] Denlinger C.S., Barsevick A.M. (2009). The Challenges of Colorectal Cancer Survivorship. J. Natl. Compr. Cancer Netw. JNCCN.

[8] Varghese C., Wells C.I., Bissett I.P., O’Grady G., Keane C. (2022). The Role of Colonic Motility in Low Anterior Resection Syndrome. Front. Oncol..

[9] Emmertsen K.J., Laurberg S. (2012). Low Anterior Resection Syndrome Score: Development and Validation of a Symptom-Based Scoring System for Bowel Dysfunction After Low Anterior Resection for Rectal Cancer. Ann. Surg..

[10] Clavien P.A., Barkun J., De Oliveira M.L., Vauthey J.N., Dindo D., Schulick R.D., De Santibañes E., Pekolj J., Slankamenac K., Bassi C. (2009). The Clavien-Dindo Classification of Surgical Complications: Five-Year Experience. Ann. Surg..

[11] Benson A.B., Venook A.P., Al-Hawary M.M., Arain M.A., Chen Y.-J., Ciombor K.K., Cohen S., Cooper H.S., Deming D., Garrido-Laguna I. (2020). NCCN Guidelines Insights: Rectal Cancer, Version 6.2020: Featured Updates to the NCCN Guidelines. J. Natl. Compr. Cancer Netw..

[12] Glynne-Jones R., Wyrwicz L., Tiret E., Brown G., Rödel C., Cervantes A., Arnold D. (2017). Rectal Cancer: ESMO Clinical Practice Guidelines for Diagnosis, Treatment and Follow-Up. Ann. Oncol..

[13] Croese A.D., Lonie J.M., Trollope A.F., Vangaveti V.N., Ho Y.-H. (2018). A Meta-Analysis of the Prevalence of Low Anterior Resection Syndrome and Systematic Review of Risk Factors. Int. J. Surg..

[14] Hughes D.L., Cornish J., Morris C., on behalf of the LARRIS Trial Management Group (2017). Functional Outcome following Rectal Surgery—Predisposing Factors for Low Anterior Resection Syndrome. Int. J. Color. Dis..

[15] Ekkarat P., Boonpipattanapong T., Tantiphlachiva K., Sangkhathat S. (2016). Factors Determining Low Anterior Resection Syndrome after Rectal Cancer Resection: A Study in Thai Patients. Asian J. Surg..

[16] Bondeven P., Emmertsen K.J., Laurberg S., Pedersen B.G. (2015). Neoadjuvant Therapy Abolishes the Functional Benefits of a Larger Rectal Remnant, as Measured by Magnetic Resonance Imaging after Restorative Rectal Cancer Surgery. Eur. J. Surg. Oncol. EJSO.

[17] Hain E., Manceau G., Maggiori L., Mongin C., Prost À La Denise J., Panis Y. (2017). Bowel Dysfunction after Anastomotic Leakage in Laparoscopic Sphincter-Saving Operative Intervention for Rectal Cancer: A Case-Matched Study in 46 Patients Using the Low Anterior Resection Score. Surgery.

[18] Carrillo A., Enríquez-Navascués J.M., Rodríguez A., Placer C., Múgica J.A., Saralegui Y., Timoteo A., Borda N. (2016). Incidencia y caracterización del síndrome de resección anterior de recto mediante la utilización de la escala LARS (low anterior resection score). Cir. Esp..

[19] Sturiale A., Martellucci J., Zurli L., Vaccaro C., Brusciano L., Limongelli P., Docimo L., Valeri A. (2017). Long-Term Functional Follow-up after Anterior Rectal Resection for Cancer. Int. J. Color. Dis..

[20] Li X., Fu R., Ni H., Du N., Wei M., Zhang M., Shi Y., He Y., Du L. (2023). Effect of Neoadjuvant Therapy on the Functional Outcome of Patients with Rectal Cancer: A Systematic Review and Meta-Analysis. Clin. Oncol..

[21] Sun R., Dai Z., Zhang Y., Lu J., Zhang Y., Xiao Y. (2021). The Incidence and Risk Factors of Low Anterior Resection Syndrome (LARS) after Sphincter-Preserving Surgery of Rectal Cancer: A Systematic Review and Meta-Analysis. Support. Care Cancer.

[22] Ye L., Huang M., Huang Y., Yu K., Wang X. (2022). Risk Factors of Postoperative Low Anterior Resection Syndrome for Colorectal Cancer: A Meta-Analysis. Asian J. Surg..

[23] Nicotera A., Falletto E., Arezzo A., Mistrangelo M., Passera R., Morino M. (2022). Risk Factors for Low Anterior Resection Syndrome (LARS) in Patients Undergoing Laparoscopic Surgery for Rectal Cancer. Surg. Endosc..

[24] Kauff D.W., Koch K.P., Somerlik K.H., Heimann A., Hoffmann K.P., Lang H., Kneist W. (2011). Online Signal Processing of Internal Anal Sphincter Activity during Pelvic Autonomic Nerve Stimulation: A New Method to Improve the Reliability of Intra-Operative Neuromonitoring Signals: Online Signal Processing of Internal Anal Sphincter Activity. Color. Dis..

[25] Filips A., Haltmeier T., Kohler A., Candinas D., Brügger L., Studer P. (2021). LARS Is Associated with Lower Anastomoses, but Not with the Transanal Approach in Patients Undergoing Rectal Cancer Resection. World J. Surg..

[26] Yan M., Lin Z., Wu Z., Zheng H., Shi M. (2022). A Predictive Nomogram Model for Low Anterior Resection Syndrome after Rectal Cancer Resection. ANZ J. Surg..

[27] Brescia A., Muttillo E.M., Angelicone I., Madaffari I., Maggi F., Sperduti I., Gasparrini M., Osti M.F. (2022). The Role of Indocyanine Green in Laparoscopic Low Anterior Resections for Rectal Cancer Previously Treated with Chemo-Radiotherapy: A Single-Center Retrospective Analysis. Anticancer Res..

[28] Ahmad N.Z., Abbas M.H., Khan S.U., Parvaiz A. (2021). A Meta-Analysis of the Role of Diverting Ileostomy after Rectal Cancer Surgery. Int. J. Color. Dis..

[29] Niu L., Wang J., Zhang P., Zhao X. (2020). Protective Ileostomy Does Not Prevent Anastomotic Leakage after Anterior Resection of Rectal Cancer. J. Int. Med. Res..

[30] Mu Y., Zhao L., He H., Zhao H., Li J. (2021). The Efficacy of Ileostomy after Laparoscopic Rectal Cancer Surgery: A Meta-Analysis. World J. Surg. Oncol..

[31] Vogel I., Reeves N., Tanis P.J., Bemelman W.A., Torkington J., Hompes R., Cornish J.A. (2021). Impact of a Defunctioning Ileostomy and Time to Stoma Closure on Bowel Function after Low Anterior Resection for Rectal Cancer: A Systematic Review and Meta-Analysis. Tech. Coloproctology.

[32] Parnasa S.Y., Chill H., Helou B., Cohen A., Alter R., Shveiky D., Mizrahi M.I., Abu-Gazala A., Pikarsky J., Shussman N. (2022). Low anterior resection syndrome following rectal cancer surgery: Are incidence and severity lower with long-term follow-up?. Tech. Coloproctology.

[33] He S., Zhang J., Wang R., Li L., Shi L., Ren D., Wang J., Deng Y., Dou R. (2022). Impact of long-course neoadjuvant radiation on postoperative low anterior resection syndrome and stoma status in rectal cancer: Long-term functional follow-up of a randomized clinical trial. BJS Open.

